# Shadow banking: a bibliometric and content analysis

**DOI:** 10.1186/s40854-021-00286-6

**Published:** 2021-10-01

**Authors:** Ridoy Deb Nath, Mohammad Ashraful Ferdous Chowdhury

**Affiliations:** Department of Business Administration, Shahjalal University of Science and Technology, Sylhet, Bangladesh

**Keywords:** Shadow banking, Review study, Bibliometric analysis, Content analysis, VOSviewer

## Abstract

This study reports on our systematic review of 2008–2021 literature on shadow banking. We present an overview of the shadow banking sector, wherein we discuss the definitions, evolution, functions, and specific activities that comprise it. We conducted a bibliometric analysis using the VOSviewer bibliometric tool on articles collected from the Scopus database, after which we conducted content analysis on top articles from leading sources, and identified four major streams of shadow banking literature. Additionally, we identified gaps in the literature and proposed seven research questions to be addressed in future studies to advance knowledge of the shadow banking sector. The findings of this review may serve as a robust reference for scholars researching various aspects of shadow banking to develop our understanding of this sector.

## Introduction

Shadow banking (SB) has been an essential and largely disputed issue in finance literature for over a decade. Its macroeconomic implications and institutional-level importance have made it a fascinating area of study for researchers and business professionals. After the Global Financial Crisis of 2007–2008 (GFC), scholars, legislators, and business professionals had SB in their sights. Many scholars have argued that the GFC initially sprouted from the SB sector, and that SB was the main culprit in the crisis (Gorton and Metrick 2010; Pozsar et al. 2010; Ashcraft and Adrian 2012; Acharya et al. 2013; Ban and Gabor 2016). The SB sector’s vulnerability was in its financing of risky, long-term, and illiquid assets with short-term borrowings, resulting in a breakdown in the credit market that forced SBs to sell long-term holdings at fire-sale prices (Geithner 2008). However, some studies argue that SBs were not entirely responsible for the sub-prime mortgage crisis during the GFC. Moreover, SBs may be a key to mitigating damages if a liquidity crisis caused by traditional banks arises in the future (e.g., Wallison 2012; Culp 2013; Culp and Neves 2017). There is also evidence that the SB system provides commercial banks with sources for increased loanable funds and assumes some of the risks associated with loan origination (Culp and Neves 2017). As the future of SB and the new financial innovations within the SB sector hangs in the balance, some suggest taming the wild horse, and others suggest letting it roam free. To that point, Wallison (2012) argued that the diverse financial innovation that is SB could be regulated away, leaving us with boring banking. On the other hand, some say that if SB remains unregulated or is regulated differently than the traditional banking sector, it will bring about the next global financial crisis (e.g., Moosa 2017). Clearly, proponents and detractors of SB have much to debate in the literature.

Our intention was to capture the diversity of positions and arguments in literature on SB. Accordingly, our main objectives were to examine the structure and development of SB research, explore the major research streams, and summarize the current state of knowledge in the field. Furthermore, we present inconsistencies in prior studies and possible explanations for them. Finally, we identify gaps in the SB literature and address needs for future research. We conducted bibliometric and content analysis on the SB articles we collected. The bibliometric analysis revealed the popular most keywords and the most influential studies, authors, and sources, along with various other aspects of SB research. Our content analysis identified four distinct research streams in SB literature, and we present important arguments from each such stream. That is, we first systematically analyzed the relevant literature retrieved from the Scopus database in our bibliometric analysis, and then, in our content analysis, we reviewed the most necessary and pertinent documents, as identified in the bibliographic analysis. This study contributes to SB literature because few review studies on SB literature exist. This review is unique in that it presents both bibliometric and content analysis of SB research from 2008 to 2021, and it further contributes to the field by identifying seven research questions that should be addressed in future studies on SB.

The remainder of the essay advances as follows: in "An overview of SB" section, we present a general review and the definitions, evolution, and functions of the SB sector, followed by a description of the study’s "Methodological approach" in second section and the "Bibliometric analysis" findings in third section. In fifth section, we present "Content analysis", describe each of the research streams, and summarize the articles most critical to each stream. Section "Content analysis" also features the seven future research questions and a thorough guide to them. Finally, in "Conclusion" section, we conclude with thoughts on the SB sector and further research opportunities in the SB literature.

## An overview of SB

### Definitions of SB

The term “shadow banking” was coined by PIMCO’s Paul McCulley, an economist and money manager, at an economic symposium arranged by the Federal Reserve Bank of Kansas City in Jackson Hole, Wyoming in 2007 (McCulley 2007). McCulley (2007) defined the SB system as “the whole alphabet soup of levered up non-bank investment conduits, vehicles, and structures.” Pozsar et al. (2010) defined SBs as “financial intermediaries that conduct maturity, credit and liquidity transformation without access to central liquidity or public sector credit guarantees.” As Noeth and Sengupta (2011) noted, the meaning of the SB banking system and its scope are widely debated in SB literature. Even the Financial Stability Board (FSB), an international body, defines the SB system from both broad and narrow perspectives. The FSB’s broad definition of SB is “credit intermediation involving entities and activities outside the regular banking system” (FSB 2011a). The FSB’s (2011a) narrow definition of SB is “(1) developments that increase systemic risk (in particular maturity/liquidity transformation, imperfect credit risk transfer and/or leverage), and/or (2) indications of regulatory arbitrage that is undermining the benefits of financial regulation.” This broad definition has been narrowed to target specific types of entities and activities.

According to Ağırman et al. (2013), SBs are a “wide myriad of highly leveraged non-deposit-taking institutions that lend long and borrow short in liquid markets.” Kodres (2013) characterized SB entities by “a lack of disclosure and information about the value of their assets (or sometimes even what the assets were); opaque governance and ownership structures between banks and shadow banks; little regulatory or supervisory oversight of the type associated with traditional banks; virtually no loss-absorbing capital or cash for redemptions; and a lack of access to formal liquidity support to help prevent fire sales.” *The *Economist (2014) put it more simply, arguing that SB should include “any bank-like activity undertaken by a firm not regulated as a bank.” Thus, we can observe the development of definitions and perspectives on SB alongside the evolution of the actors participating in SB over the last 14 years. Ultimately, as Pozsar (2018) argued, what SB is and which actors are involved in it may vary depending on whom we ask.

### Evolution of SB

According to McCulley (2007), the SB system originated with the birth of Money Market Mutual Funds (MMMFs) in the 1970s. Some authors also reported the “emergence of an unregulated parallel banking system” (presumably SB) in the early 1990s (D'Arista and Schlesinger 1993). Gorton and Metrick (2010) attributed the development of the SB system to the regulatory and juridical changes that indulged three types of institutions: “MMMFs to capture retail deposits from traditional banks, securitization to move assets of traditional banks off their balance sheets, and repurchase agreements (repos) that facilitated the use of securitized bonds as money.” Others are of the view the SB system originated to fill a gap in the economic system (Landau 2019).

**Fig. 1:**
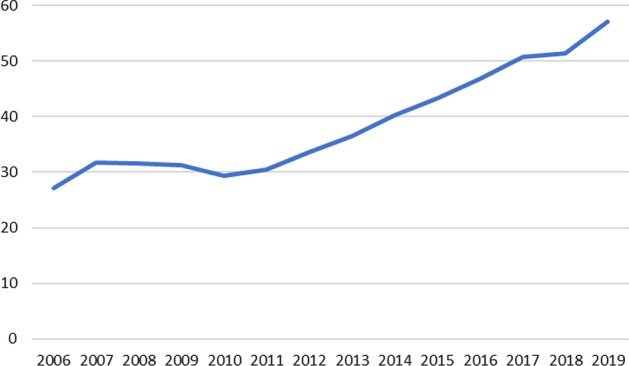
Shadow banking assets in the World (USD trillion). *Source*: FSB (2020)

Whatever the case, the size of SB has increased rapidly since the GFC. For instance, FSB (2020) reveals that the narrow measure of SB assets increased by 11.1% to 57.1 trillion (USD) in 2019 from the previous year, representing 14.2% of total global financial assets. Figure 1 depicts the growth of shadow banking assets (narrow measure) from 2006 to 2018.

**Fig. 2:**
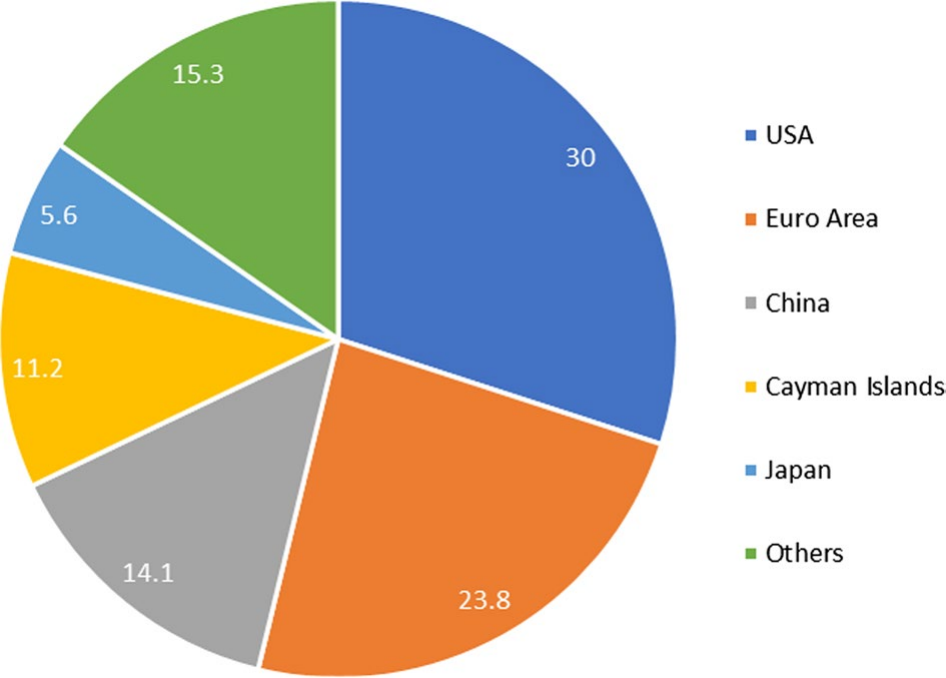
Share of shadow banking assets by region (%). *Source*: FSB (2020)

Additionally, the FSB report reveals the global share of SB assets (narrow measure) by the 29 jurisdictions it monitors. Figure 2 depicts the percentage of SB assets in 2019 by the 29 jurisdictions.

Here, the Euro Area includes Belgium, France, Germany, Ireland, Italy, Luxembourg, Netherlands, and Spain. Other areas include the other FSB 29-group countries.

### Functions and activities of SB

SBs execute credit intermediation functions between investors and borrowers and generate income in this process from the difference in interest rates or fees. However, SBs are not subject to a similar level of regulatory requirements as traditional banks (Moosa 2017). Accordingly, Gorton and Metrick’s (2010) argued that “fundamental changes in the financial system in the last 30–40 years, as a result of private innovation and regulatory changes, together led to the decline of the traditional banking model.” They further argued that as traditional banks encountered severe competition from the non-bank financial institutions sector, they too sought new profit opportunities in the shadows.

As noted above, the FSB defines SB narrowly and broadly when measuring its scope. The broad measure comprises the non-bank financial intermediation (NBFI) sector and is also known as the Monitoring Universe of Non-bank Financial Intermediaries (MUNFI) (FSB 2019). MUNFI encompasses all financial institutions that are not banks, central banks, or other public financial institutions. Thus, the FSB’s broad measure of SB includes pension funds, insurance companies, and other financial institutions (OFIs). The OFI subset includes finance companies, investment funds, trust companies, captive financial institutions and money lenders (CFIMLs), structured finance vehicles, and the like.

The FSB’s narrow definition of SB (presented in Table 1 with examples) is based on economic functions.

**Table 1:** FSB’s narrow definition of shadow banking and example of classified entity type

Economic functions	Definition	Typical entities
Economic-function: 1	Management of collective investment vehicles with features that make them susceptible to runs	MMFs, fixed-income funds, mixed funds, credit hedge funds, real estate funds
Economic-function: 2	A loan provision that is dependent on short-term funding	Finance companies, leasing/factoring companies, consumer credit companies
Economic-function: 3	Intermediation of market activities that is dependent on short-term funding or secured funding of client assets	Broker-dealers, securities finance companies
Economic-function: 4	Facilitation of credit creation	Credit insurance companies, financial guarantors, monoline
Economic-function: 5	Securitization-based credit intermediation and funding of financial entities	Securitization vehicles, structured finance vehicles, asset-backed securities

*Source*: FSB (2020)

Some traditional banking areas are strongly interconnected with SB entities. Traditional banks engage in SB activities through off-balance-sheet accounts (e.g., Tian et al. 2016; An and Yu 2018; Tymoigne and Wray 2013) by supplying credit money to enterprises using non-standard accounting measures that bypass regulatory constraints (Sun 2019). “Shadow or money creation by banks beyond traditional loans” has two main components: (1) assets channeled by other traditional banks (e.g., reverse repo of bills, dual buyout of credit assets, interbank payments, etc.) and (2) activities channeled by non-bank financial intermediaries (e.g., credit swaps, trust beneficial interests, asset management plans, etc.) (Sun 2019). Although SB lending is similar to traditional bank lending, shadow loans are not listed as loans on the balance sheet. Such activity is often listed as interbank assets, investment assets, or off-balance sheet items. FSB (2011b) states that traditional banks extend financial support to the SB sector by granting loans, investing in SB products, or involving themselves in the SB intermediation process. Banks can also own finance companies and other SB entities (FSB 2012).

## Methodological approach

Our approach to reviewing SB literature combined bibliometric analysis and content analysis. The bibliometric analysis method has been frequently adopted in literature review studies (e.g., Iddy and Alon 2019; Naatu and Alon 2019; Bahoo et al. 2019; White et al. 2016). The content analysis method is widely used in business and finance literature (e.g., Zha et al. 2020; Paul and Rosado-Serrano 2019; Paul and Benito 2018; Paul and Singh 2017). The same combined methods approach was previously adopted by Bahoo et al. (2020a, b).

**Fig. 3:**
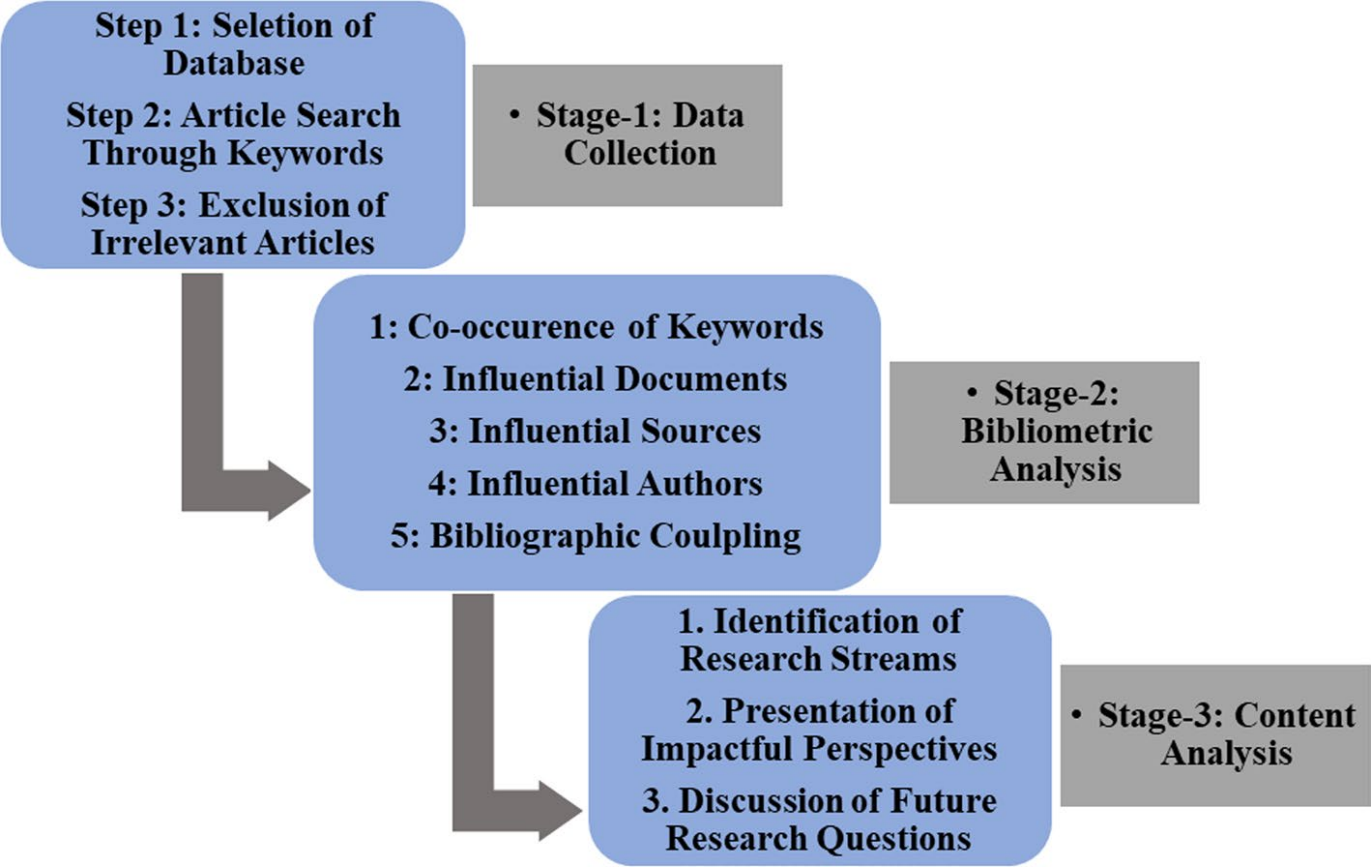
Workflow of the literature review process

The literature review process used in this study is depicted in Fig. 3. We conducted the review in three stages. In the first stage, we collected 419 articles returned from the search term “shadow banking” from the SCOPUS database, chosen because it is the largest database of peer-reviewed literature (Zhang et al. 2020; Chadegani et al. 2013). We customized the SCOPUS Excel file, and, after screening the articles based on title and relevance, found 185 articles in the SB branch of financial literature. To ensure that no critical articles were skipped, and to confirm that screened-out articles were, in fact, irrelevant to our research, we surfed the SCOPUS journals on finance manually.

The second stage of our review process was dedicated to bibliometric analysis of the 185 articles, using VOSviewer software. VOSviewer is a bibliometric analysis tool developed by Van Eck and Waltman (2010) to construct and view bibliometric maps. It can display such maps in multiple ways, each of which projects different aspects. This tool has been previously used by Donthu et al. (2020), Feng et al. (2020), Gutiérrez-Nieto and Serrano-Cinca (2019), Niñerola et al. (2019), Castillo-Vergara et al. (2018). We use it to analyze six aspects of SB literature. In the third stage of the review process, we conducted content analysis and manually identified four distinct research streams based on topical keywords and careful reading of abstracts. For additional specific details on the processes, which are, for purposes of clarity, covered along with the results, see the sections that follow.

## Bibliometric analysis

### Bibliometric findings

We analyzed the co-occurrence of all keywords used in the SB literature. We also examined the most highly cited documents, authors, and sources. Furthermore, we applied bibliographic coupling to determine shared sources between articles. The results of the bibliometric findings are presented in the following sections.

#### Co-occurrence of keywords

As the field of shadow banking literature is well-diversified, and there are some 372 of keywords frequently used in the literature over time, we filtered them by imputing five as the minimum number of occurrences of a keyword, and 14 of the 372 keywords met the threshold.

**Fig. 4:**
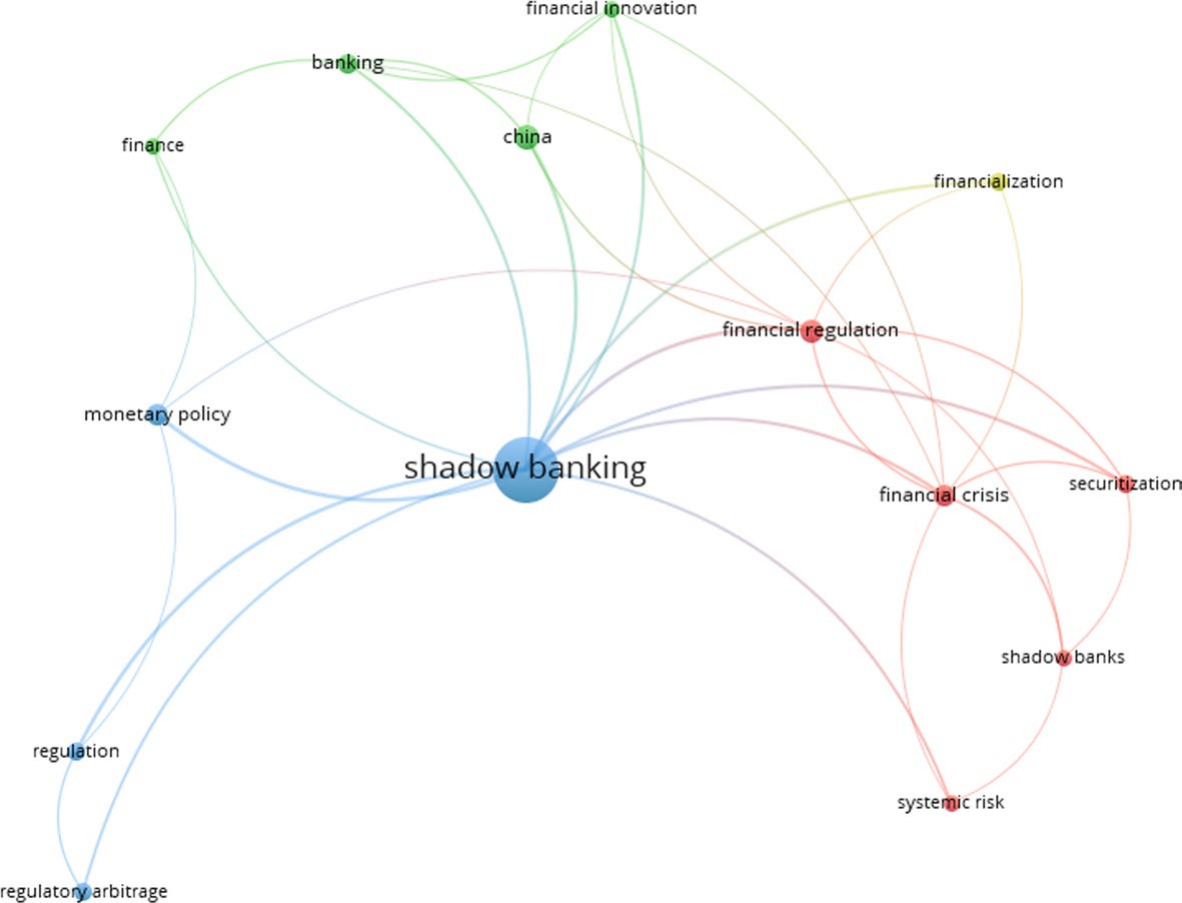
Network map of co-occurrence of all keywords. *Source*: Authors’ own estimation

In Fig. 4, the node size represents the magnitude of the keyword’s occurrence (Krauskopf 2018; van Eck and Waltman 2014). The figure thus indicates that the term “shadow banking” was the most frequently used of the 14 keywords identified. Other frequently used terms include “monetary policy,” “China,” “financial regulation,” “regulation,” “financial crisis,” “shadow banks,” “regulatory arbitrage,” and “systemic risk.” The results further revealed that the strongest link was between the terms “shadow banking” and “monetary policy.” Additionally, “shadow banking” was found to have strong links with “regulation,” “financial regulation,” and “China.” These findings suggest that the monetary policy and regulation of the financial system are the most common concerns regarding the SB sector.

#### Most influential documents

To determine the most highly cited documents in SB literature, we filtered the analysis to return documents that were cited a minimum of 15 times. Twenty of the 185 articles meet the threshold. Table 2 presents those 20 highest cited documents in SB literature.

**Table 2:** Highest cited documents on SB

References	Number of citations	References	Number of citations
Gorton and Metrick (2010)	146	Adrian and Shin (2010)	24
Gennaioli et al. (2013)	85	Acharya et al. (2013)	22
Adrian and Shin (2010)	65	Ban et al. (2016)	22
Rixen (2013)	48	Bengtsson (2013)	21
Plantin (2015)	37	Li (2014)	19
Chernenko and Sunderam (2014)	36	Gabor (2016)	19
Lu et al. (2015)	35	Nesvetailova (2015)	19
Lysandrou and Nesvetailova (2015)	34	Chen et al. (2018)	17
Sunderam (2015)	31	Kodres (2013)	15
Stein (2010)	31	Tsai (2015)	15

*Source*: Authors’ own estimation

**Fig. 5:**
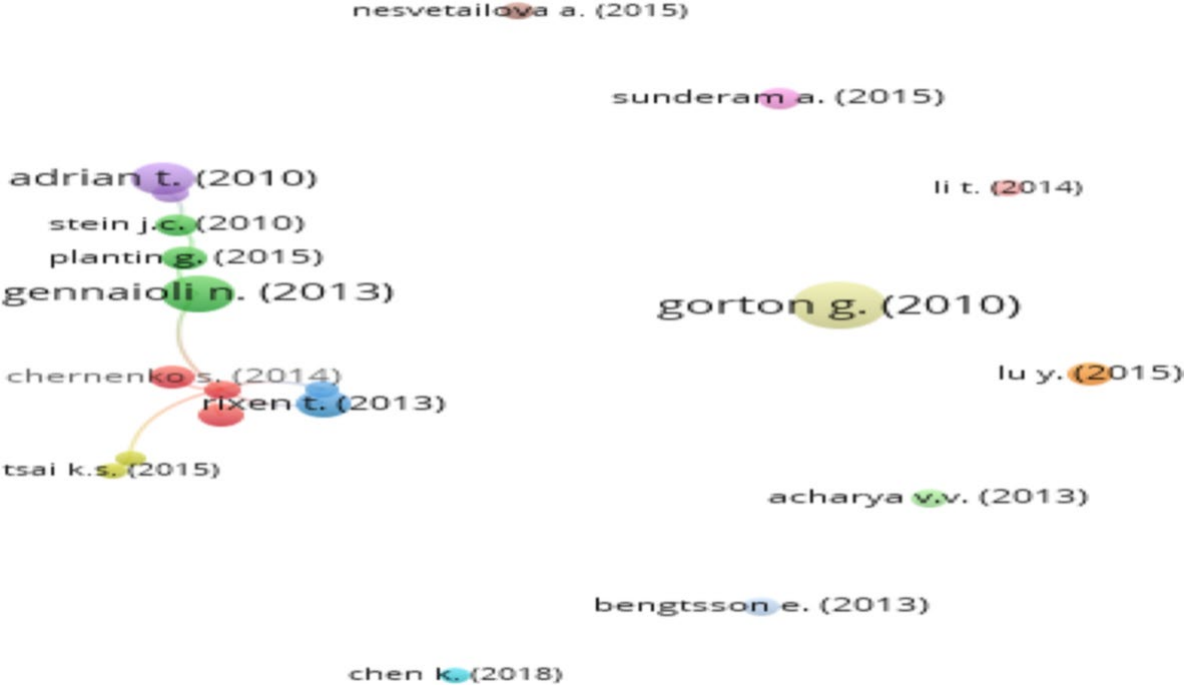
Network map of the highest cited documents on SB. *Source*: Authors’ own estimation

Figure 5 presents the network map based on the most highly cited articles.

We found that a paper by Gorton and Metrick (2010) is the most highly cited document; however, it is not connected to the set of 12 interconnected articles as shown in Fig. 5. Similarly, Bengtsson (2013), Acharya et al. (2013), Li (2014), Lu et al. (2015), Nesvetailova (2015), Sunderam (2015), and Chen et al. (2018) are also absent in the connected set, despite being among the 20 most cited documents.

#### Most influential sources

This section reveals the most highly cited sources in the field of SB literature and the number of documents that rely on each such source. Here, to filter the sources, we selected one as the minimum number of documents for a source and 16 as the minimum number of citations to a source. After filtering, 15 of the 121 sources were returned. Table 3 presents a summary of those most influential sources.

**Table 3:** Highest cited sources with corresponding number of documents

Source	Documents	Citations
Brookings Papers on Economic Activity	1	146
Review of Financial Studies	3	104
Journal of Finance	2	98
Review of International Political Economy	7	88
Annual Review of Economics	1	65
International Review of Economics and Finance	7	49
Regulation and Governance	1	48
Journal of International Money and Finance	3	43
Daedalus	1	31
Annual Review of Financial Economics	2	26
Journal of Financial Economic Policy	2	23
New Political Economy	2	22
Journal of European Public Policy	1	19
American Economic Review	1	17
China Economic Review	2	16

*Source*: Authors’ own estimation

#### Most influential authors

**Fig. 6:**
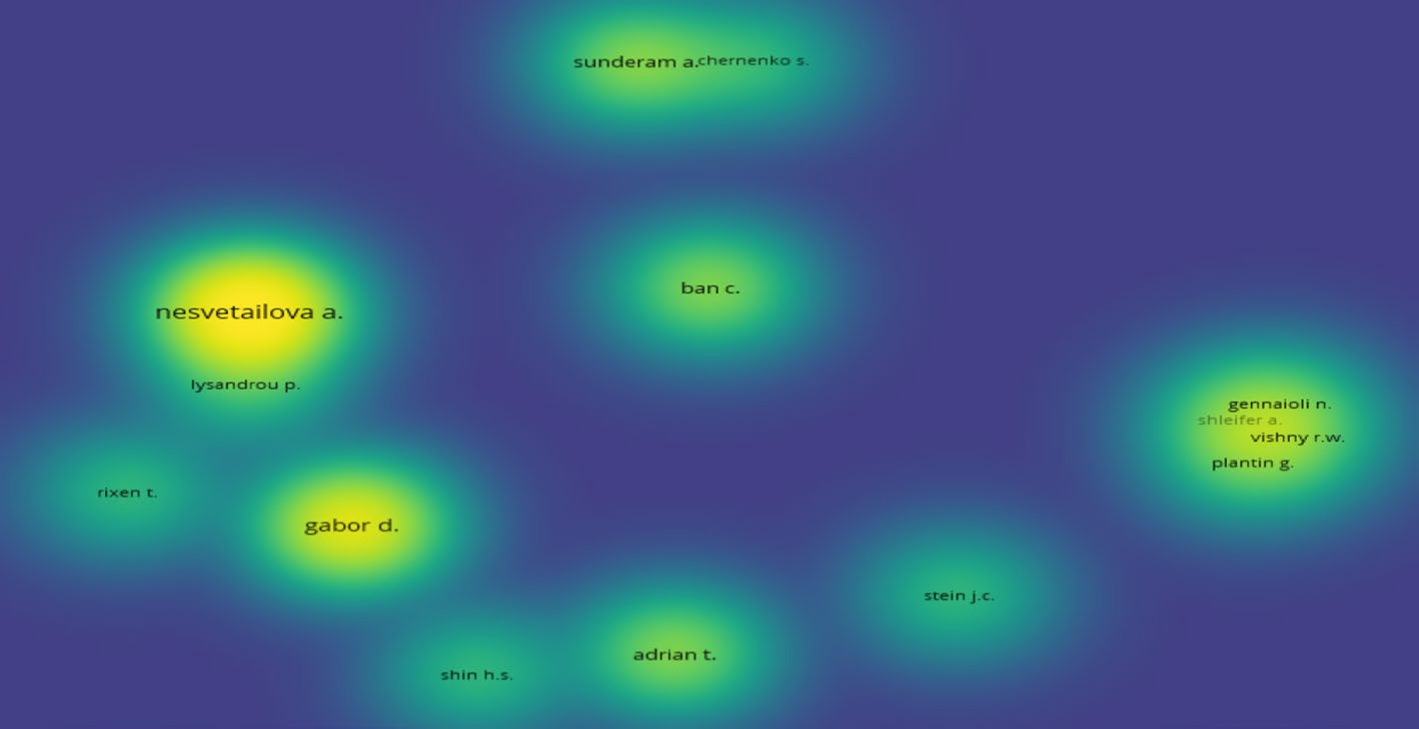
Density visualization of authors with the highest number of documents. *Source*: Authors’ own estimation

To identify the most influential authors in SB literature of shadow banking, we considered a maximum of 5 authors per document during the filtration process. Further, we used the minimum number of documents one and the minimum number of citations as 30 for filtration purposes. Twenty of the 284 authors met the threshold. Figure 6 presents a density visualization featuring the authors with the highest number of documents in the shadow banking literature.

Our findings suggest that Anastasia Nesvetailova published the most documents on SB, followed by Daniela Gabor. However, Gary Gorton and Andrew Metrick (146 citations each) were the most highly cited authors in the field. The following four authors are, as follows (documents, citations), Tobias Adrian (2,89), Nicola Gennaioli (1,85), Andrei Shleifer (1,85), Robert W. Vishny (1,85).

#### Bibliographic document coupling

**Fig. 7:**
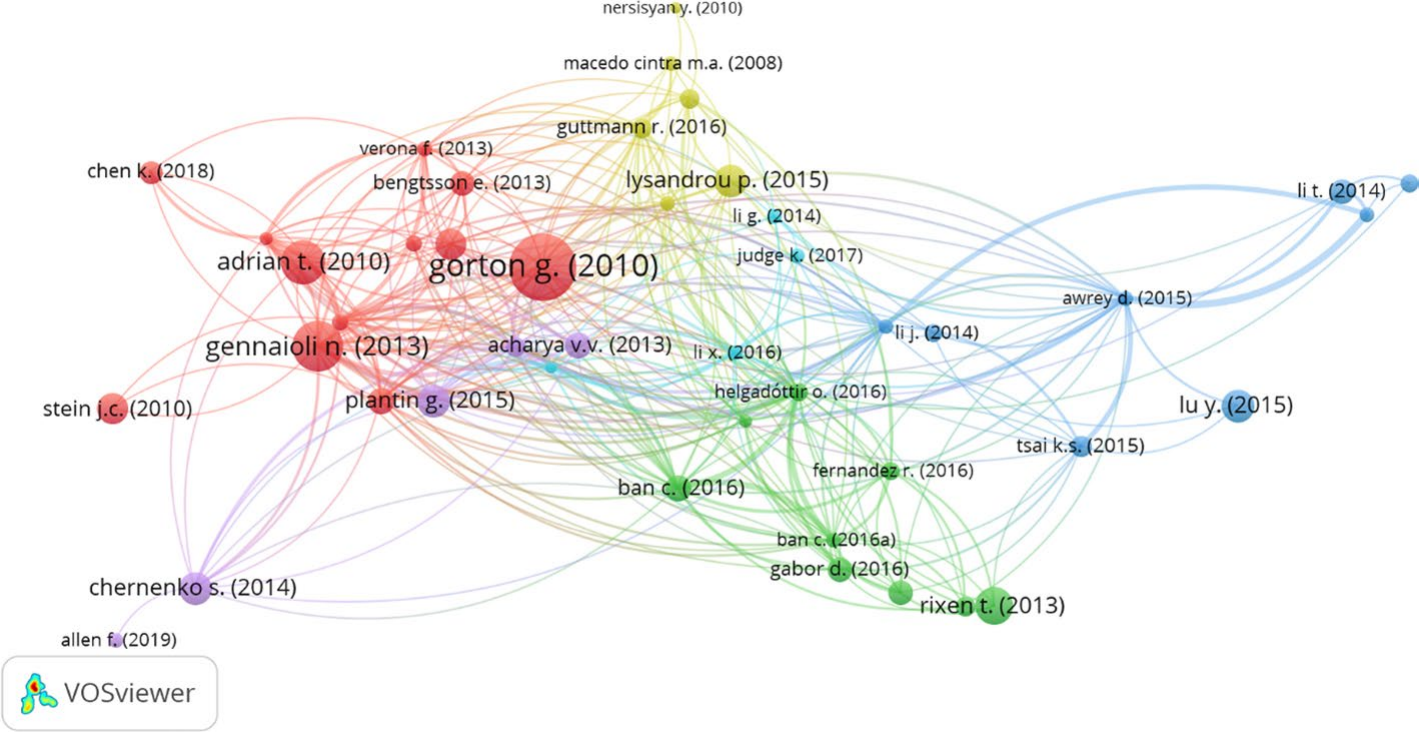
Network visualization of bibliographic coupling of documents. *Source*: Authors’ own estimation

The developers of VOSviewer suggest that bibliographic coupling results represent the overlap of references between publications. The greater the number of shared references between two papers, the greater the strength of the link between them. Here, we filtered the analysis by inputting the minimum number of citations of a document as 5, resulting in 47 articles. However, only 44 articles were found in the connected set. Visualization of the bibliographic coupling of documents in the SB network is presented in Fig. 7.

Helgadóttir (2016) was revealed as the document with the most significant total bibliographic coupling link strength (84 with 11 citations). The other top 5 articles ranked as follows. For each of the articles, the first number stands for total link strength and the second number for the number of citations. Moreira and Savov 2017) [75,13], Meeks et al. (2017) [72,9], Ban et al. (2016) [66,22], Awrey (2015) [66,7], Gennaioli et al. (2013) [66,85].

## Content analysis

### Major research streams

During content analysis, we analyzed the documents based on their relevance to our discussion and ensured that no critical document was skipped in the discussion. We identified four major research streams in SB literature using the topical keywords identified in the prior studies and found in the abstracts of the subject studies. Categorizing the research streams assists in visualizing the dimensions of SB that have been studied over the course of its evolution. However, we acknowledge that some articles belong to multiple streams because of the multidisciplinary nature of SB studies. Summaries of the main arguments in each stream are presented in Tables 4, 5, 6, and 7. Description of the articles and their findings are presented in the following four sections.

**Table 4:** Key literature on determinants of SB

References	Journal/conference	Country/region	Main arguments/findings
Apostoaie and Bilan (2020)	Economic Research-Ekonomska Istraživanja	Central and Eastern European countries	Economic growth and institutional investor’s higher funding demand positively affect the development of the shadow banking sector. Additionally, investors depend on shadow banks for higher yields in a low-interest rate situation
Barbu et al. (2016)	Review of Economics and Business Studies	15 European countries	Stock market indices and the long-term interest rates positively influence the shadow banking size, while the development of investment funds and the M2/GDP ratio negatively impact
Hodula et al. (2017)	European Financial Systems 2017	Spain	An increase in term spreads and low interest rates tend to affect the growth of shadow banking positively. Moreover, the country-specific characteristics and individual components of shadow banking have due importance in such studies
Zhou and Tewari (2019a)	Cogent Economics & Finance	14 emerging economies and Singapore	A negative relationship exists between shadow banking and monetary policy. Shadow banking increases when bank risk-taking is reduced
Hodula et al. (2020)	Economic Systems	24 EU countries	Difficile financial development, strict regulation, and demand for long-term institutional investors positively influence the shadow banking growth
Kim (2017)	IFC Conference, Bank for International Settlements	G-20 Countries	Insurance companies and pension funds positively influence shadow banking growth. The size of banks’ assets also reveals similar results
Duca (2016)	Journal of Banking & Finance	USA	Change in information and reserve requirements costs and shift in bank-nonbank credit sources regulation has a negative and long-run impact on shadow banks’ share in funding short-term business debt. This share fell in the short run when short-term liquidity premia, term premia, and event risks in the security market increased

**Table 5:** Key literature on SB and systemic risk

References	Journal/conference	Nature	Main arguments/findings
Pellegrini et al. (2017)	Finance Research Letters	Empirical	MMFs listed in the UK have decreased systemic risk during GFC. Average systemic risk is increased by liquidity mismatch over the whole study period, but the risk only decreases during GFC
Bengtsson (2016)	Journal of Financial Regulation and Compliance	Theoretical	From the perspective of systemic risk, hedge funds and conventional investment funds have several commonalities. Investment funds’ ability to substitute traditional banks’ maturity transformations may be threatened by the instability in funding profiles of the investment funds
Wymeersch (2017)	EBI Working Paper Series	Theoretical	Banks have been bound to adapt the behaviors, structure, and balance sheet to the risk of participating in the shadow banking market
Hsu et al. (2013)	CITYPERC Working Paper Series	Theoretical	Several factors, including herd behavior, are creating systemic risk in the European markets. Risk dispersion across the underdeveloped segment of the shadow banking sector has posed China some concentrated risks that have no contribution to the systemic risk
Tian et al. (2016)	Emerging Markets Finance and Trade	Empirical	Over the 2007–2012 period, trust companies posed the most financial Instability in China and this adverse effect caused the commercial banks the most
Wei (2015)	Asia Pacific Law Review	Theoretical	Regulators and the market players object to shadow banks’ existence as it generates financial risks. However, commercial banks have enjoyed a large amount of profit generated by the WMPs. So, the Chinese policymakers can formulate a multi-tiered market for loans and an interest rate environment to cure WMPs’ systemic risk
Hsu et al. (2014)	PERI Working Paper Series	Empirical	Trust companies pose the most systemic risk in the Chinese shadow banking system. The systemic risk posed by banks, insurance companies, and securities companies had insignificant differences. Banks absorbed the most systemic risks, about 85%, in the shadow banking system

**Table 6:** Key literature on policy and political issues of SB

References	Journal/conference	Nature	Main arguments/findings
Yang et al. (2019)	Pacific-Basin Finance Journal	Empirical	Shadow banking has a negative influence on welfare at times of monetary policy shock. Coordinating leverage ratio regulations and monetary policies would help stabilize the financial system and decrease the size of shadow banking
Zhang and Wan (2017)	Emerging Markets Finance and Trade	Empirical	Chinese economic activities need to be guided by a mix of policy instruments. The transition from quantitative policy tools to other price-based instruments will not be easy, but it will capture the monetary policy stances to a little extent, especially for the open market operations
Nesvetailova (2015)	New Political Economy	Theoretical	Shadow banking results from regulative arbitration in the traditional banking system enhanced by the nationwide accounting, taxation, and banking rules. The administrative arbitration approaches towards shadow banking are useful but limited
Hou et al. (2018)	International Review of Economics and Finance	Empirical	Political intervention negatively impacts the bank cost efficiency, which weakens the positive relationship between bank cost efficiency and shadow banking growth
Fève et al. (2019)	Journal of Economic Dynamics & Control	Empirical	As the macroprudential policies only target the traditional banks and the sector leakage reduces their effectiveness, wider regulations addressing shadow credit may help stabilize the economy
Bryan et al. (2016)	Review of International Political Economy	Theoretical	The shadow banking sector is not only a sector of erratic financial practice and a reason for financial fragility but also a sector of political and juridical innovation
Ban and Gabor (2016)	Review of International Political Economy	Theoretical	Redistributing wealth on a large scale is a better solution to redress the economic structural imbalance than better regulations of shadow banks. Thus, the responsibility is mostly on the influencers of income inequality and not on the regulatory authority

**Table 7:** Key literature on SB and financial stability

References	Journal/conference	Nature	Country(s)/region	Main arguments/findings
Barth et al. (2015)	Journal of Financial Economic Policy	Theoretical	China	Shadow banks may prove useful by enhancing greater savings and investment opportunities and diversifying the Chinese financial sector
Bengtsson (2013)	Journal of International Money and Finance	Theoretical	Europe	Transparency makes it difficult for European MMFs investors to distinguish between MMFs based on asset quality. Also, policy coordination needs to be improved when unusual steps are taken to protect financial stability
Bouguelli (2020)	Journal of Post Keynesian Economics	Theoretical		As ‘financial layering’ increases in the economy alongside the development of shadow banking, financial fragility increases too. Additionally, as a large part of shadow banking is an alternative source of funds for banks, financial fragility increases alongside the development of shadow banking
Culp (2013)	Journal of Applied Corporate Finance	Theoretical		In the leveraged loan market, bank syndicates heavily rely on non-bank investors. Therefore, the existence and non-existence of these investors marginally affect the banks’ ability to extend C&I credits
Culp and Neves (2017)	Journal of Applied Corporate Finance	Theoretical	United States	The overall risk exposure of commercial banks is being diversified to the non-bank sector. At times of liquidity crisis, shadow banking can help commercial banks mitigate their short-term funding needs
Liang (2016a)	The Chinese Economy	Theoretical	China	Although the shadow banking system in China is considered a helpful complement to the traditional banking sector, it poses risks to the wider financial system. Therefore, reform in the financial system is required as shadow banking develops
Tsai (2016)	The Journal of Development Studies	Theoretical	China	China’s SMEs suffer from a financing gap, and shadow banking keeps filling it with credit supply in the market
Liang (2016b)	Journal of Economic Issues	Theoretical	China	Shadow banks engage in business activities that increase institutional risks. Although shadow banks promote credit-driven financial growth, such growth makes the financial system fragile
Diallo and Al-Mansour (2017)	Research in International Business and Finance	Empirical	(Multiple)	When the shadow banking system was used as a channel, the insurance sector was found to be harmful to the financial stability of a country where shadow banking assets were large
Landau (2019)	SEACEN Financial Stability Journal	Theoretical		The development of shadow banking is related to the need to fill a gap in the financial system. Although the shadow banking system is complex, sophisticated, and potentially dangerous, it is also necessary
Zhou and Tewari (2019b)	Cogent Economics & Finance	Empirical	South Africa	In South Africa, shadow banking negatively affects traditional banks’ profitability. However, it positively impacts the profitability of non-financial firms. Additionally, it has a positive impact on aggregate firm profitability
Peter Watkins (2011)	International Journal of Productivity and Performance Management	Theoretical	Canada	The adoption of shadow banking has proven beneficial to the labor productivity matrix over a large period
Zou et al. (2013)	Quality & Quantity	Empirical	China	The shadow banking system in China develops the overall financial system. Nevertheless, the excessive growth of shadow banking makes the economy fragile. Therefore, the shadow banking system should progress slowly and under the light of the regulatory body
Ilesanmi and Tewari (2019)	Cogent Economics & Finance	Theoretical		Shadow banking in South Africa is beneficial as it provides alternative sources of credit and extends investment opportunities for the economy. However, a lack of transparency, management, and regulations poses a great risk to the economy

#### Determinants of SB

A limited number of studies have addressed the determinants of SB during the study period. However, the unique nature of these articles requires categorizing them separately within a distinct research stream.

Several older studies explained the process by which regulatory requirements, such as reserve requirements, give rise to alternatives to bank loans (e.g., Kanatas and Greenbaum 1982; Bernanke and Lown 1991; Duca 1992; Berger and Udell 1994) and to securitization (Pennacchi 1988). Other studies have also noted that changes in information costs contribute to the long-run rise of SB (e.g., Edwards and Mishkin 1995; Ratnovski 2013), although empirical assessment has been only rarely conducted. The first empirical study on determinants of SB was conducted by Barbu et al. (2016) and covered Austria, Belgium, Finland, France, Germany, Greece, Hungary, Italy, Latvia, Luxembourg, Norway, Romania, Slovenia, Spain, and Sweden. In that study, the dependent variable SB was proxied by the net value of the assets of monetary funds. The independent variables were the real GDP variation, interest rate level (both short-term and long-term), M2/GDP ratio, GDP contribution of investment funds’ assets, and levels of stock market indices. Barbu et al. (2016) noted linear relationships between SBs and various macroeconomic indicators. Their findings suggest positive relationships between an SB’s size and long-term interest rates and stock market indices. However, they observed negative relationships between an SB’s size and short-term interest rates, investment fund growth, GDP growth, and M2/GDP ratio.

Duca (2016) addressed the impact of capital regulation and other factors on the role of SB in funding short-term business debt. Their findings reveal that changes in information and reserve requirement costs and shifts in bank-nonbank credit source regulation had a negative and long-run impact on shadow banks’ share in funding short-term business debt. Additionally, their share fell in the short-run when short-run liquidity premia, term premia, and event risks in the security market increased. However, it rose again when the economic outlook improved, risk premia declined, and deposit interest rate ceilings were more binding. Kim (2017) conducted a dynamic panel estimation on G-20 countries to identify factors that drive shadow banking in an economy. He reported that insurance companies and pension funds positively influenced SB growth. Kim (2017) additionally argued that the size of banks’ assets had a positive and significant relationship with SB, as suggested by the originate-to-distribute model. Hodula et al. (2017) conducted another study on shadow banking in Spain with SBs’ assets as the dependent variable, using the broad measure of SB. Traditional banks’ assets, term spread, real GDP, and interest rates (short-term) were independent variables. Hodula et al. (2017) argued that the traditional banking sector’s size positively influenced the shadow banking sector’s size, and, in most cases, the shadow banking sector reacted pro-cyclically to the development of Spain’s real GDP. Hodula et al. (2017) furthermore found that an increase in term spread and low-interest rates positively influenced the growth of the Spanish SB sector. Apostoaie and Bilan (2020) conducted a study on 11 central and eastern European countries, taking both the broad and narrow measures of SB as dependent variables. Independent variables comprised real GDP growth rate, term spread, the growth rate of the total financial assets of pension funds and insurance corporations, money market interest rate, growth rate of the total reserves (excluding gold), and the growth rate of the total assets reported by the Monetary Financial Institutions (as defined by the European Central Bank). Apostoaie and Bilan (2020) found that developments in the traditional banking sector, institutional investors sector, and money market interest rate, overall liquidity and economic conditions influence the SB sector positively. Term spread, however, was found to negatively influence the SB sector. Apostoaie and Bilan (2020) also argued that the development of the SB sector complements the development of the overall financial system. Zhou and Tewari (2019a) investigated SB, bank risk-taking, and monetary policy nexus. They reported a negative relationship between SB and monetary policy. However, they argued that as shadow banking increased, banks reduced their risk-taking in the market. Additionally, they reported a positive relationship between SB and GDP and real effective exchange rates and that inflation negatively affected SB growth. The latest study conducted by Hodula et al. (2020) revealed that stiff financial development, strict regulation, and demand for long-term institutional investors positively influenced SB growth. However, they also reported that factors influencing SB growth may function differently in different countries. Other studies reported a “search for yield” effect, i.e., that investors looked for high-yielding assets in the SB sector (e.g., Goda et al. 2013; Lysandrou 2011).

Table 4 presents the main arguments in key literature on determinants of SB.

#### Shadow banking and systemic risk

Studies on systemic risk and SB are likewise limited but important enough to merit a stream. Some such studies analyzed SB’s overall systemic risk exposure (e.g., Wymeersch 2017; Hsu et al. 2013). Others addressed specific components of SB and the systemic risk exposure that each entailed (e.g., Wei 2015; Bengtsson 2016; Pellegrini et al. 2017).

Bengtsson (2016) analyzed the systemic risk implications of investment funds from a theoretical standpoint. His is the earliest systemic risk study on the role of investment funds in SB. He (2016) distinguished three main systemic risk features on a theoretical basis and considered the possibility of their interconnectedness in real financial system scenarios. He posited that such interconnectedness could increase systemic risk in the credit intermediation process. He further argued that, from the perspective of systemic risk, although hedge funds and conventional investment funds are quite different in terms of business models, they have several commonalities; moreover, investment funds’ ability to substitute traditional banks’ maturity transformations could be threatened by instability in investment funds’ funding profiles. Huang (2018) modeled a continuous-time macro-finance framework where shadow banking is viewed as the off-balance-sheet financing of traditional banks. Huang (2018) suggested that shadow banking is pro-cyclical and that it increases endogenous risks. Pellegrini et al. (2017) studied systemic risk implications of money market funds listed in the UK. Their findings suggest that such funds lowered rather than elevated systemic risk during the GFC. Further, Pellegrini et al. (2017) argued that average systemic risk was increased by liquidity mismatch over the whole study period, but that risk only decreased during the GFC. In contrast, ECB (2020) predicted that in the unprecedented COVID-19 turmoil, the stress in MMFs could spill over to MMF-reliant sectors to manage their liquidity and constrain the financial system and real economy from access to liquidity and short-term funding. However, IOSCO (2020) suggested that MMF category, currency, and strategy differences should be carefully considered when comparing the GFC to the recent stress faced by MMFs.

Tian et al. (2016) empirically investigated systemic risk in China’s SB system at the sector level. They included traditional banks, trust companies, securities companies, fund management companies, and insurance companies and found that trust companies were “the main culprit” in China’s financial instability. They further found that commercial banks assumed significant risks in the SB system over the 2007–2012 period. In that vein, Tian et al. (2016) found that commercial banks went around regulators’ reserve deposit ratio and adequacy rate policies by conducting off-balance-sheet transactions, increasing systemic risk in the market. Wei (2015) conducted another study on the Chinese SB system, theorizing the implications of wealth management products on systemic risk. SB, he explained, is considered the black market by regulators and market players. Accordingly, regulators and market players object to its existence on the basis of the financial risks that it generates. However, Wei (2015) argued that such players in the form of commercial banks have enjoyed significant profit generated by wealth management products. He suggested that, accordingly, Chinese policymakers should formulate a multi-tiered market for loans and an interest rate environment based on the market to cure systemic risk in wealth management products.

Hsu et al. (2014) studied the systemic risk implications of banks, securities companies, insurance companies, trust companies, and fund management companies in China on China’s SB system. Similar to Tian et al. (2016), they found that trust companies comprise the most significant portion of systemic risk in China’s SB sector. Hsu et al. (2014) also found insignificant differences in the systemic risk levels posed by banks, insurance companies, and securities companies in the SB sector. They confirmed Tian et al.’s (2016) findings that banks absorbed the most systemic risk, about 85%, in the SB system. Wymeersch’s (2017) article on systemic risk and shadow banks theorized that banks were compelled to adapt their behaviors, structures, and balance sheets to the risks of acting in the SB market. Wymeersch (2017) also analyzed several regulations targeting SBs and found that in most such regulations, the motives to protect investors were inexistent. In Hsu et al.’s (2013) study on SB in Europe and China, several factors, including herd behavior, were found to have created systemic risk in European markets. Furthermore, risk dispersion across the underdeveloped segment of the SB system has led China to some concentrated, localized risks. However, such a segment was not found to contribute to systemic risk (Hsu et al. 2013).

The main arguments found in key literature on SB and systemic risk are presented in Table 5.

#### SB politics, policies, and the regulation debate

Before the GFC, regulators and academics remained unaware of the SB sector to a great extent, highlighting the suitability of the term “Shadow Banking” to refer to it (Helgadóttir 2016). The SB sector does not operate under the safety umbrella of a central bank and comprises many divisions that function independently and, for the most part, lies outside the strict regulations and standard monetary policy that the traditional banking sector is subject to, such as reserve requirements. In 2014, *The Economist* warned that because it is a large, almost unknown, and fast-growing industry, SB could become “a global bogeyman.” They also predicted that although SB could be used as a solid mechanism to avert the next financial crisis, if not managed carefully, it could be of severe detriment to the world economy. There is some tension between that idea and Wallison’s (2012) assertion that regulation could cost us this diverse “financial innovation” and leave us with “boring banking,” which we saw early on in this review. The political debate among regulators and academics, which addresses the future of SB, including applicable regulations and monetary policies, is an evolving one and one to watch. Accordingly, we turn to SB studies in the fields of law and economics.

An emerging consonance between academic literature and legislative literature is a vision of SB as the result of regulative arbitration in the traditional banking system enhanced by national accounting, taxation, and banking rules (Nesvetailova 2015). Hachem (2018) argued that stringent liquidity regulations led to the rise of SB in China. As seen above, many have argued that SBs will bring about the next global financial crisis if left unregulated (e.g., Moosa 2017). Wullweber (2020) found that central banks were adapting to this new financial innovation and were in the process of providing ample reserves and access to their balance sheets to SB participants. However, Wullweber (2020) also acknowledged that such policies created contradictions and fragilities and that central banks were searching for other possible ways of dealing with this banking innovation.

Bengtsson (2013) and Plantin (2015) argued that relaxing capital requirements may be optimal if conventional banks can bypass it in the SB system. In a recent study, Irani (2020) concluded that tightening banks’ capital regulation gives rise to non-bank presence. They added that when banks with weak capitalization reduce loan exposure, mostly by loan sales, the non-bank sector picks up the slack. Moreover, Zhang (2020) argued that when the extensive-margin effect outweighs the intensive-margin effect, stringent capital requirements will induce rather than curb a credit boom. Zhang (2020) also argued that both total loans and real GDP would be negatively impacted by raising capital requirements if the debt market was segmented and borrowers did not have the option to choose between commercial banks and SBs. Ordonez (2013) proposed combining conventional regulations and cross reputation subsidization to increase sustainability in the SB sector since banks are more concerned for their reputations than SBs. In this regard, Yang et al. (2019) argued that the SB sector is perhaps a strong amplification tool that weakens the implementation of monetary policies and decreases the effectiveness of macroeconomic policies. Further, they added, in times of technological shock, loan-quota shock, and bank’s net-worth shock, SB positively influences welfare; conversely, in times of loan-to-deposit ratio shock and monetary policy shock, shadow banking negatively influences welfare. However, Yang et al. (2019) also argued that using look-through regulations to improve financial stability may negatively impact the economy. Therefore, they concluded that coordinating leverage ratio regulations and monetary policies would help stabilize the economy but would also reduce the size of the SB sector. On the other hand, in their study on the Chinese economy, Zhang and Wan (2017) argued that Chinese economic activities should be guided by a mix of policy instruments and that a transition from quantitative policy tools to other price-based instruments would not be easy but would capture monetary policy stances to some extent, especially for open market operations. Nesvetailova (2015) also argued that administrative arbitration approaches to SB are useful but limited considering the scope, diversity, and complexity of SB participants. Wu and Shen (2019) presented evidence suggesting that banks involved in SB activities take more risks than those that are not, but added that good governance could reduce this effect. That being the case, as the macroprudential policies only target traditional banks, and sector leakage reduces their effectiveness, wider regulations addressing shadow credit may help stabilize the economy (Fève et al. 2019).

Political intervention is yet another piece of the puzzle. Hou et al. (2018) found that political interference weakens managers’ ability to efficiently manage financial intermediaries and make optimal production decisions based on market information. Furthermore, evidence from that study suggests that political intervention negatively impacts bank cost efficiency, thus weakening the positive relationship between bank cost efficiency and shadow banking growth. Despite that position, some regulation tools have already been proposed, and others are already being implemented. The Dodd-Frank Act passed in the US in 2010 introduced specific provisions that brought hedge funds, OTC derivatives, retail lenders, and various other institutions important to the SB system under regulation. Gorton and Metrick (2010) argued, however, that the Dodd-Frank act has too many regulatory gaps and proposed the establishment of narrow savings banks and narrow funding banks for regulating reorganization and securitization. Shleifer and Tarullo (2010) argued against Gorton and Metrick (2010), suggesting that their proposal would significantly restrict all ABSs. As policymakers become more concerned with the SB and newer insights into the SB sector emerge, the policy and regulatory debate will surely evolve further, presenting new confluences and conflicts. Table 6 presents the main arguments of the key literature addressing policy and political issues of SB.

#### SB and financial stability

This section explores the implications of SB on financial stability and financial development. SB and its implications on financial stability and financial development are of great concern to regulatory authorities. The SB sector was not properly addressed before the GFC, and since then, numerous financial innovations have been taking place within the SB sector.

In the early 1990s, the “emergence of an unregulated parallel banking system” (SB) was observed by some authors D'Arista and Schlesinger 1993). After the GFC, shadow banking came to the attention of the major regulatory bodies as it was often considered the main culprit in the crisis. Some researchers even argued that the crisis sprouted from the SB sector (Pozsar et al. 2010; Gorton and Metrick 2010; Adrian and Ashcraft 2012; Acharya et al. 2013; Ban and Gabor 2016). Others argued that SB was not entirely to blame for the 2007–2008 sub-prime mortgage crisis and that SBs may be keys to mitigating future liquidity crises in the financial system (e.g., Wallison 2012; Culp 2013; Culp and Neves 2017). Moreover, Wullweber (2020) argued that a stable financial system is currently dependent on a stable SB system.

Shadow banking has recovered quickly since its collapse in 2007–2008, and it outgrew its size (broad measure), capturing an increasing share of the global GDP in 2015, according to FSB (2016). Additionally, the SB sector is increasing rapidly worldwide. Bengtsson (2013) has argued that non-bank financial intermediaries, otherwise known as SBs, and their implications on financial stability must be better understood to alleviate or even avert the next global financial crisis. The growth of SB and the inconclusiveness of the debate on SB prospects make studies addressing its implications on financial stability crucial. Some of the studies addressing such implications focused on specific components of SB (e.g., Culp 2013; Bengtsson 2013), and others addressed the overall SB sector (e.g., Barth et al. 2015; Liang 2016a, Tsai 2016; Zhou and Tewari 2019a, b; Ilesanmi and Tewari 2019). Bengtsson (2013) conducted a study on the European economy and reported that there is a lack of transparency regarding the composition of Money Market Fund (MMFs) assets making it difficult for European MMF investors to distinguish between MMFs based on their asset quality. Additionally, Bengtsson (2013) suggested that policy coordination must be improved when unusual measures are taken to protect financial stability. It is noteworthy that several regulatory authorities have already been established to allow for close and effective policy coordination between all relevant agents regularly and during times of crisis. For example, the European Systemic Risk Board was established in 2010 in Europe to prevent and mitigate systemic risk.

Sun (2019) stated that banks’ shadow activities increase their credit risk and that they do not adequately assess that risk or take preventive measures. Bouguelli (2020) theorized how shadow banking operates and affects the overall market in the process. Bouguelli (2020) concluded that as “financial layering” increases in the economy alongside the development of shadow banking, financial fragility increases as well. SBs can fatally interrupt financial market equilibrium and inject instability into the financial system that regulators will need to control (Erturk 2017). Furthermore, in the leveraged loan market, bank syndicates rely heavily on non-bank investors, suggesting that whether or not these investors exist marginally affects the banks’ ability to extend C&I credits (Culp 2013). Additionally, as a large part of SB is an alternative source of funds for banks, financial fragility increases alongside the development of SB (Bouguelli 2020). However, the SB system does provide sources for increased loanable funds for commercial banks and assumes some of the risks associated with the loan origination. Therefore, portions of commercial banks’ overall risk exposure are being diversified to the non-banking sector. If the next financial crisis occurs in reverse where traditional banks suffer from a liquidity crisis, SBs can be key in mitigating short-term funding shortages in the financial system (Culp and Neves 2017). Additionally, evidence suggests that money demand functions are becoming more stable with the emergence of SB (Serletis and Xu 2019).

Nevertheless, Huang (2018) presented SB as the off-balance-sheet financing of traditional banks and reported that a limited level of risk-sharing does not improve financial stability. Barth et al. (2015) conducted a study on the Chinese economy and stated that if the Chinese SB system encounters difficulties, such difficulties may also affect China’s commercial banks. However, China’s regulatory body has developed several policies for governing SB operations and the interconnectedness of SBs and commercial banks. Furthermore, evidence suggests that more municipal, corporate bonds were issued during 2012–2015 in the provinces that saw greater bank loan growth in 2009, along with the development of SB activities (Chen et al. 2020). Additionally, as China’s SMEs suffer financing gaps, SB keeps filling these gaps with the supply of credit to the market (Tsai 2016). As such, SBs may prove useful in China by promoting greater and enhanced savings and investment opportunities and diversifying the Chinese financial sector (Tsai 2016, Barth et al. 2015). Additionally, empirical evidence shows that SB positively impacts Chinese economic growth, money supply, and interest rates in the market (Gabrieli et al. 2018).

Zhu (2021) suggested that SB activities induced by conventional banking before 1996 increased efficiency and helped drive credit to more profitable non-state sectors. However, Zhu (2021) reported that the positive effects of SB were limited to real estate investments and that the effects on private firms outside this sector were negative. Moreover, Liang (2016a), in his study on China, reported that although the SB sector in China is considered a helpful complement to the Chinese traditional banking sector, it poses risks to the wider financial system.

Loose regulations and SBs’ institutional characteristics allow SBs to engage in business activities that increase institutional risk. Although SBs promote credit-driven financial growth, such growth increases the fragility of the financial system (Zou et al. 2013; Liang 2016b). Another study, this one on the South African economy, revealed that SB negatively affected traditional banks’ profitability. Furthermore, Ari et al. (2017) argued that the liquidation of SBs could leave traditional banks susceptible to liquidity risk when the SB sector’s size is large. However, large size positively impacts the profitability of non-financial firms. It was additionally found that the SB sector positively impacts aggregate firm profitability (Zhou and Tewari 2019a, b). Another study argued that SB in South Africa is beneficial as it provides alternative sources of credit and extends economic investment opportunities; yet, lack of transparency, management, and regulations poses great risk to the economy (Ilesanmi and Tewari 2019). In contrast, a study on the Canadian economy suggested that Canada’s productivity problem could be solved through innovation. Financial innovation, in this aspect, may come as an SB system. Additionally, the adoption of SB has proven beneficial to Canada’s labor productivity matrix over a long period (Watkins 2011). Moreover, Landau (2019) argued that the development of SB relates to the need to fill gaps in the financial system. Therefore, although the SB system is complex, sophisticated, and potentially dangerous, it is also necessary.

Table 7 presents the main arguments made in the key literature on SB and financial stability.

### Discussion and future research questions

The literature on SB is maturing rapidly, and it covers a wide range of research areas. Nonetheless, some aspects of SB remain inconclusive and vastly explorable. Accordingly, we here contribute to the study of SB. We identified seven research questions derived from our exploration of SB literature that should be undertaken in future research to further advance knowledge and understanding of SB.

#### What drives SB in economies?

SB is growing rapidly, both in developed and developing economies. It recovered quickly from its 2007–2008 collapse and outgrew its size (broad measure) in terms of its share of the global GDP in 2015, according to the FSB (2016). However, the forces driving economies towards SB activities are yet to be clearly defined in the literature. The limited number of studies on the topic suggests scope for further exploration of factors leading to the development of SB. As suggested by Apostoaie and Bilan (2020) and Hodula et al. (2017), the impact of regulative arbitrage opportunities on SB could be addressed in this regard. Consistent with Kou et al. (2021a), our study suggests that country-specific analyses can provide essential recommendations for improving the financial systems of particular countries. Simultaneously, we acknowledge that analyses of region interconnectedness can generate interesting findings, as SB participants can be registered in one region and operate in another and can offer financial products to regions on a global scale. That being said, we do agree with Hodula et al. (2017) that systematically breaking down and analyzing the different components of SB may present more meaningful and more specific results.

#### Which components of SB pose the most systemic risk?

Systemic risk caused by SB is of great concern to regulatory authorities. However, the question of which shadow sector components pose the greater systemic risk to the economy is not addressed thoroughly in the literature. Studies analyzing the systemic risk implications of specific SB components could strengthen our understanding of SB and the risks associated with it. Allen et al.’s (2019) findings from a study on entrusted loans in China have several implications for how that component of SB influences the traditional banking sector and the economy at large. Pellegrini et al. (2017) addressed the systematic risks posed by UK MMFs and suggested that analyses of other components of SB, such as finance companies, are likely to yield additional relevant findings in this regard. As An and Yu (2018) stated in their article analyzing off-balance sheet activities in China, the SB sector is highly diversified, and multiple components are largely neglected. Furthermore, the need for studies on the quantification of SB widens the scope for research in this aspect, as different measurement tools reveal different sizes and proportions of components of the SB sector. Moreover, efforts should be made to compile a superior and more comprehensive database. For example, additional detailed breakdowns of investment fund portfolios may allow us to view microscopic components of these shadow entities. Ultimately, SBs’ overall systemic risk exposure is yet to be addressed properly in the literature and requires scholarly attention. Indisputably, scholarly contributions can be increased by developing granular and anonymized databases and making them publicly available.

#### How should SBs be regulated?

An SB regulation system is another area yet to be properly addressed in the literature. The results of debate among scholars and regulators regarding the existence and regulation of the SB sector are inconclusive. Many scholars suggest that SB participants that do not fall under the regulatory umbrellas and safety nets of central banks should be eradicated (e.g., Bouguelli 2020). Others suggest that the SB sector should be regulated more effectively according to its unique properties so that the benefits of SB can be enjoyed without leading to greater systemic risk in the economy (e.g., Ilesanmi and Tewari 2019). Furthermore, policy coordination should be improved when unusual measures are taken to protect financial stability, as suggested by Bengtsson (2013). More effective supervision of the interconnectedness between the SB sector and the traditional banking sector is also called for to prevent systemic financial layering and ensure the fulfillment of liquidity requirements. Equally importantly, interconnectedness among the different participants in the SB sector should be closely supervised within a macroprudential framework designed to avoid contagion and spillover in both normal times and crises. There is a growing need for studies addressing regulatory reforms and prescriptions, and these will likely be highly appreciated by scholarly and regulatory entities.

#### What are the implications of SBs on financial stability?

Despite accusations of generating financial instability on various occasions, SB is growing rapidly worldwide. The implications of SBs on overall global financial stability remain inconclusive in SB literature. Mixed results and contradictory theories present evidence both in support of and against this sector. All of these can be considered key initiators of research addressing SBs’ implications on financial stability. As noted, many argue that SBs increase financial instability in the market by acting as an alternative source of funds for commercial banks and by creating “financial layering” in the market or by interrupting financial market equilibrium (e.g., Erturk 2017; Bouguelli 2020). Łasak et al. (2019) reported that traditional banking and SB are interconnected at three levels (layers): banks’ off-balance-sheet financing, indirect support for banks’ lending, and non-bank lending. Most post-Keynesians have suggested eliminating or constraining shadow banks to the greatest extent possible. Others have suggested that assuming that another financial crisis will not occur in the traditional banking sector can prove fatal (Landau 2019). In the future, SBs could be key in mitigating liquidity crises in the traditional banking sector (Culp and Neves 2017). Thus, it is important to further research which parts of the SB system, under which types of shocks, can generate financial instability.

#### How should systemic risk of SB be measured?

As we have seen, the levels of systemic risk associated with the development of SB activities is another matter of great concern to academics and regulators alike. However, absolute and specific risk measurement tools have not heretofore been developed to measure the systemic risk posed by SBs, and conventional risk measurement tools may not reflect the realities of the SB sector. To that end, Ilesanmi and Tewari (2019) suggested that a unique systemic risk measurement tool be developed for SBs. There appears then to be a growing need for studies suggesting and implementing unique systemic risk measurement tools for SBs.

#### How does SB development impact traditional banks?

SBs and traditional banks are highly interconnected in the financial system. Empirical studies and theoretical discussions on how SBs impact traditional banks present contradictory results and remain up for debate among academics. As we have seen, multiple studies argue that the traditional banking sector is under threat from the SB sector and that the SB sector reduces traditional banks’ profitability (e.g., Zhou and Tewari 2019a, b; Ding et al. 2020), while others present evidence of a complementary relationship between SBs and traditional banks in the economy (Liang 2016a). SBs provide alternative funding sources for traditional banks and assume some of the risks associated with loan origination. SBs may also provide traditional banks with liquidity support at times (Culp and Neves 2017). As we have seen, traditional banks may move assets off balance sheets, although there is no legitimate reason for them to do so (Tymoigne and Wray 2013), suggesting that the impact of SBs on traditional banking sector requires additional scholarly attention.

#### What risks and vulnerabilities does the SB system face?

As SBs are major players in the world economy, their risks and vulnerabilities can pose risks and introduce vulnerabilities in the greater economy. Moreover, if SBs were to acknowledge the risks and vulnerabilities they face, such a move might likewise disrupt the overall economy. Fang et al. (2020) did address China’s entrusted loan market and empirically tested loan risk and collateral in the SB system. However, what kinds of risks SBs may face and what they may be vulnerable to remain to be covered in the literature. Tasky (2019) has done some work in this regard, identifying liquidity risks and risks related to leverage, interconnectedness, and contagion, and to procyclicality, leverage, and liquidity. Tasky (2019) also pointed to significant gaps in data on SBs. Moreover, studies similar to Kou et al. (2021b) should be conducted to develop bankruptcy prediction models for SB entities. Addressing the risks and vulnerabilities of SB entities may bring important and significant findings to this evolving debate.

## Conclusion

Our current endeavor employed a bibliometric tool to trace the evolution and development of SB literature. It identified and presented the most recognized knowledge in the area of SB and attempted to synthesize the key theoretical and empirical findings on SB. VOSviewer, the bibliometric tool employed, is scientific, credible, and has been previously applied in practice by Donthu et al. (2020), Feng et al. (2020), Gutiérrez-Nieto and Serrano-Cinca (2019), Niñerola et al. (2019), to name a few, as noted. Our methods thus reflect the trend in SB research, and our findings can be deemed credibly derived.

After identifying the major papers, sources, and authors in SB literature, content analysis was applied to categorizes the material into four distinct streams, which are discussed in context, with summaries of the key arguments.

However, this study is limited in that the Scopus database was the sole source of the data for bibliometric analysis. Including other databases (e.g., Web of Science, Dimensions) will allow for more thorough mapping of the SB research network. Additionally, more rigorous analyses of individual SB components and addressing interconnectedness among SB entities in different regions will move us toward more finely-tuned insights. We further acknowledge that limiting the data gathered to the results returned from a single search query “shadow banking” may have hindered the presentation of an exhaustive view of the sector, as many articles address the sector without applying the terminology. The research can be expanded in the future by including additional search terms such as “systemic risk,” “non-bank financial intermediaries,” and “market-based finance.” Furthermore, to keep pace with the rapid evolution of SB literature and the SB sector itself, similar studies should be conducted periodically to update the results and continue fine-tuning our understanding.

Growing innovation in the field of finance is contributing to the complexity of the SB sector, and researchers are addressing these newer developments in their studies. Additionally, the COVID-19 pandemic has resurrected some previous concerns about SB. Crisis scenarios can accelerate the growth of SB through firms’ increasing demand for alternative sources of financing. Moreover, as the interest rates are predicted to remain low over the next several years, investors will turn to more profitable alternatives, contributing to the growth of assets in the SB sector. In contrast, decreases in money circulation in the market induced by the abrupt declines in economic activities due to COVID-19 and related issues can also force the halt of development in the SB sector. Accordingly, much more research is required to understand and broaden our knowledge of SB. Our study model can be extended in the future through the use of multiple databases and different bibliometric tools and by reviewing the SB sector by specific economic region. We further suggest that debates on SB entities’ systemic risk exposure and vulnerabilities cannot be concluded until sufficient evidence and additional comprehensive studies are undertaken, and their findings revealed. We suggest that addressing the seven questions presented in the present study in future research questions will move us to a deeper and more accurate understanding of the SB sector and to more informed conclusions on best practices for managing this financial innovation for national and global welfare.

## Data Availability

Data used in this paper were collected from Scopus database.
